# Revolutionary Approaches to Hair Regrowth: Follicle Neogenesis, Wnt/ß-Catenin Signaling, and Emerging Therapies

**DOI:** 10.3390/cells14110779

**Published:** 2025-05-26

**Authors:** Apoorva Mehta, Mateen Motavaf, Danyal Raza, Alison J. McLure, Kofi D. Osei-Opare, Lindsey A. Bordone, Alejandro A. Gru

**Affiliations:** 1 Vagelos College of Physicians and Surgeons, Columbia University, New York, NY 10032, USA; ajm2366@cumc.columbia.edu (A.J.M.); kdo2118@cumc.columbia.edu (K.D.O.-O.); 2 Duke University School of Medicine, Durham, NC 27710, USA; mateen.motavaf@duke.edu (M.M.); danyal.raza@duke.edu (D.R.); 3 Department of Dermatology, Columbia University Irving Medical Center, New York, NY 10032, USA; lab87@cumc.columbia.edu (L.A.B.); aag2222@cumc.columbia.edu (A.A.G.)

**Keywords:** follicle neogenesis, B-Catenin, hair regrowth, hair therapies, alopecia, alopecia areata, JAK

## Abstract

With alopecia affecting millions globally, recent advancements in the understanding of hair follicle biology have driven the development of novel therapies focused on hair regrowth. This review discusses two emerging therapeutic strategies: hair follicle neogenesis and the modulation of the Wnt/B-catenin signaling pathway. Hair follicle neogenesis, a frontier once considered impossible to achieve in adult humans, has recently gained traction due to advancements in stem cell biology and further understanding of the epithelial–mesenchymal interactions that are critical to hair follicle development. Such an approach shows significant potential for addressing conditions leading to hair loss, such as androgenetic and scarring alopecias. The Wnt/B-catenin signaling pathway, a critical intracellular pathway responsible for hair follicle cycles, has gained traction as a target for therapeutic interventions. Studies show that stimulating this pathway leads to hair follicle growth, while its inhibition prompts hair follicle regression. Investigations demonstrate clinical efficacy of small molecule inhibitors and peptides, such as PTD-DBM, which activates the Wnt/β-catenin pathway by interfering with CXXC5, a negative regulator that inhibits pathway activation. Such therapies show potential as more effective treatment options than existing solutions such as finasteride and minoxidil. Adjunctive therapies, such as low-level laser therapy, have also shown clinical efficacy, further highlighting how modulation of this pathway stimulates follicular regrowth. While these novel therapies require further research to validate their efficacy and to gain additional insight into their risk profile, it is clear that alopecia treatment is approaching a new frontier beyond traditional pharmacologic interviews, with regenerative medicine and pathway modulation paving the way forward.

## 1. Introduction

Alopecia, encompassing a broad range of hair loss disorders, is a prevalent condition that impacts millions globally. Despite its low acuity, alopecia has a significant psychosocial impact on patients, including but not limited to depression, reduced self-esteem, and quality of life, emphasizing the need for effective therapies [1,2]. Conventional treatments such as minoxidil and finasteride have shown improved outcomes for patients but their clinical efficacy and risk profile are not consistent across patients [3]. Investigators continue to develop novel interventions to fully reverse follicular loss without the risk of side effects.

Advancements in molecular and cellular biology have revealed key regulatory pathways contributing to follicular neogenesis, morphogenesis, and regeneration. A key signaling pathway is the Wnt/β-catenin cascade, which governs hair follicle stem cells and their transition into the anagen phase, the part of the follicular cycle where hair grows [4]. Researchers have begun to investigate how modulation of this pathway can result in hair follicle neogenesis, described as the de novo creation of follicles in adults, a feature that was considered impossible by many specialists [5]. Pre-clinical models involving Wnt/β-catenin pathway modulation have exhibited favorable outcomes, underscoring the therapeutic potential that may be possible in human skin.

Other cellular pathway elements have also gained traction as potential therapeutic targets. JAK-STAT inhibitors, commonly used in patients who suffer from autoimmune disease, have shown efficacy in activating dormant hair follicles in autoimmune-related hair loss conditions such as alopecia areata [6]. Tangentially, modulation of prostaglandin signaling between prostaglandin E2 (PGE2) and prostaglandin D2 (PGD2) has also been implicated in follicular cycling [7]. Other emerging therapies, such as stem cell-based approaches, growth factor delivery, and CRISPR gene editing, are being actively explored for their potential to address hair loss and promote regrowth.

This review provides a comprehensive overview of alopecia therapies, ranging from treatments for androgenetic and scarring alopecias to alopecia areata. By exploring the roles of Wnt/B-catenin signaling, hair follicle neogenesis, JAK-STAT inhibitors, prostaglandin analogs, and other novel therapies, this review aims to discuss the current state of research and translational challenges involved in developing long-term solutions to alopecia (Table 1).

## 2. Hair Follicle Neogenesis

Hair follicles are one of the most dynamic miniaturized organs humans possess. Embedded within the dermis, hair follicles contain complex anatomical structures that play fundamental roles in follicular growth and regeneration.

### 2.1. Structure and Cycling of Hair Follicles

#### 2.1.1. Anatomy of a Follicle: Dermal Papilla, Epidermal Stem Cells, and Extracellular Matrix

The dermal papilla (DP) is a mesenchymal structure located at the base of the hair follicle. Via paracrine signaling, DP cells regulate epithelial stem cells located within the bulge area and govern follicular cycling. The extracellular matrix (ECM) surrounds the hair follicle, both providing structural support and mediating growth factor signaling [8]. Epidermal stem cells are then located slightly above the follicle’s root in the bulge area. Epidermal stem cells in the bulge are unique as they can differentiate into multiple cell types, such as the outer root sheath or hair shaft [9].

#### 2.1.2. The Four Phases of Hair Cycling: Anagen, Catagen, Telogen, Exogen

Hair follicles constantly change their physiology and anatomy as they are subject to four stages: anagen, catagen, telogen, and exogen. The first cycle, anagen, is the active growth phase. During anagen, keratinocytes proliferate to create the hair shaft. It is during this phase that Wnt/B-catenin signaling is highly active [10]. The catagen phase is the involution phase in which apoptosis causes follicular regression, prompting follicular stem cell quiescence. Bone morphogenic protein (BMP) signaling is commonly noted during this phase. Follicles then remain dormant in the telogen phase until inductive signaling from dermal papilla cells. Exogen, the final phase, is the shedding phase, where old hairs fall out and new growth begins [10,11].

### 2.2. Cellular Regulation in Hair Follicle Growth

Hair follicle cycling requires precise governance through inductive signaling, cellular interactions, and signaling pathways.

#### 2.2.1. Dermal Papilla: Inductive Signaling

Dermal papilla (DP) cells are characterized as central regulators of the hair follicle cycle. They possess particular control over transition from dormant (telogen) to growth (anagen) phases. Described as mesenchymal cells, they secrete growth factors such as fibroblast growth factor (FGF) and insulin-like growth factor 1 (IGF-1) [12].

FGF7 and FGF10, in specific, play a critical role in epithelial stem cell activation and proliferation [13]. They initiate hair follicle growth by stimulating keratinocyte activity and follicular elongation. IGF-1 is also an important element contributing to healthy follicular growth by both preventing apoptosis during anagen and also triggering vascular endothelial growth factor (VEGF) expression to improve follicular blood flow [14,15].

FGFs and IGF-1 are vital due to their synergistic ability to activate quiescent epithelial stem cells in the bulge region. They promote differentiation into a fundamental structural component of a hair follicle: matrix keratinocytes [13,14]. Dysregulation of these two growth factors is frequently seen in pathologies such as androgenetic alopecia, which ultimately results in follicle miniaturization and reduced hair growth. Targeting DP dysfunction is a novel avenue that investigators are currently exploring in preclinical models.

#### 2.2.2. Epithelial–Mesenchymal Interactions in Folliculogenesis

Interactions between epithelial and mesenchymal elements of the hair follicle ultimately drive folliculogenesis. Epithelial progenitors receive intercellular signals from dermal papilla cells through pathways such as bone morphogenetic proteins (BMPs) and Hedgehog (Shh) [16].

BMP signaling ensures appropriate timing of epithelial differentiation by limiting progenitors to a quiescent state until appropriate. This spatial and temporal control prevents premature differentiation [16]. Furthermore, the Shh pathway further reinforces epithelial–mesenchymal interactions by activating downstream elements such as GLI1 [17]. The synergistic relationship between the Shh pathway and other pathways, such as Wnt/B-catenin, ensures well-synchronized follicular maturation.

These two pathways promote epithelial proliferation and hair follicle elongation. Since epithelial–mesenchymal interactions are not restricted to embryonic development, they have been highlighted as potential targets for future therapies.

#### 2.2.3. Epidermal Stem Cells: Differentiation into Follicular Lineages

Epidermal stem cells are multipotent stem cells in the bulge region that give rise to progenitor cells that differentiate into specific follicular lineages. The most important of these lineages is the matrix keratinocytes. These cells form the hair shaft, inner root sheath, and outer root sheath. Bulge epidermal stem cells express key markers such as CD34, K15, and Lgr5, which allow them to be distinguished from other epidermal progenitor cells [18].

During the growth phase, anagen, bulge epidermal stem cells mobilize and migrate down towards the hair matrix. They begin proliferating to promote follicular generation. The Wnt/B-catenin signaling pathway is a critical component of this observed behavior. Activation of Wnt signaling causes B-catenin to translocate to the nucleus and interact with transcription factors LEF-1 and TCF3 to amplify the expression of lineage-specific genes [18]. These genes promote differentiation. Inhibiting the Wnt/B-catenin pathway prompts stem cell quiescence and halts hair regeneration [19].

As epidermal stem cells proliferate at the base of the follicle, microenvironmental signals prompt them to turn into one of three cell types:

1. Matrix Keratinocytes: These cells form the shaft of the follicle and medulla. Wnt and BMP signaling trigger their creation.
2. Inner Root Sheath: This provides structural support to the shaft. It consists of the cuticle, Huxley, and Henle layers. BMP signaling and epithelial–mesenchymal interactions guide inner root sheath creation.
3. Outer Root Sheath: Epidermal stem cells also form the outer root sheath, another form of follicular structural support. The literature also points towards the outer root sheath acting as a reservoir for stem cells and the bulge area [18,19].

Furthermore, epidermal stem cell differentiation relies on dermal papilla cell signals. Cellular elements secreted by DP cells such as FGF-7 and Shh interact with Wnt signals to promote proliferation. This pathway communication ensures that proliferation is temporally and spatially controlled throughout each phase of the hair cycle [20].

The ability of epidermal stem cells to differentiate into three critical lineages makes them an attractive target for regenerative therapies. Investigators are actively exploring how the Wnt/B-catenin signaling pathway can be modulated to restore follicular function through epidermal stem cell differentiation in conditions such as androgenetic alopecia and scarring alopecias [21]. However, there are still challenges in precisely controlling stem cell fate.

### 2.3. Translational Challenges

Despite significant strides in understanding hair follicle biology, challenges remain that hinder their effectiveness in clinical settings.

#### 2.3.1. In Vitro Limitations

The microenvironment of human hair follicles is complex and difficult to replicate in vitro. It consists of an intricate balance between mesenchymal and epithelial cells, extracellular matrix components, and paracrine signaling pathways.

An example of this difficulty is with dermal papillary cells. In vivo, DP cells must be in a specific three-dimensional spatial orientation and in close communication with epithelial cells to maintain their inductive ability [22]. When removed from this environment, DP cells quickly lose their inductive abilities when cultured in monolayers. Furthermore, in vitro systems also struggle to replicate the gradient-dependent signaling of the Wnt, BMP, and Shh pathways [23]. Without accurate replication, improper stem cell activation and follicular development occur. These elements underscore the difficulty of mimicking hair follicle microenvironments in static culture systems.

#### 2.3.2. Animal Model Variability

Rodents are frequently used as animal models in preclinical research. Although they share some similarities in physiology and cell biology, rodents and humans have significant differences in dermal papilla biology.

One of these differences is the size and morphology of hair follicles. Human hair follicles are significantly larger and have many more structural elements. As a result, scaling successful findings in mouse models to clinical human trials is often met with unfavorable outcomes [24]. On a cellular level, rodent DP cells exhibit higher intrinsic plasticity and inductive potential [25]. Human DP cells instead require support from FGF and IGF-1 to maintain their inductive ability in static cell cultures. The models also differ in immune responses, as follicular transplantation outcomes and immunomodulatory interventions often fail in humans due to more robust immune system activation [26].

#### 2.3.3. Innate and Adaptive Immune Response

The innate immune system often rejects transplantation of regenerated hair follicles into the human scalp. Macrophages and dendritic cells recognize the transplanted follicles are foreign invaders and release inflammatory cytokines such as tumor necrosis factor (TNF-a) and interleukin-1 beta (IL-1B), leading to apoptosis of dermal and epithelial cells [27]. The adaptive immune response also plays a role in rejection. Antigen presentation from dendritic cells or macrophages from the innate immune system activates cytotoxic CD8+ T cells that infiltrate the transplanted tissue and target dermal papilla and epithelial stem cells. Furthermore, allogenic follicles elicit an even more aggressive immune response due to an MHC antigen mismatch [27].

#### 2.3.4. Potential Solutions

To address these multifaceted challenges, researchers are investigating the potential of immunomodulatory agents to limit rejection. Cyclosporine A, also known as tacrolimus, inhibits T-cell activation and has shown promise in reducing follicular rejection [28]. Monoclonal antibodies targeting local TNF-a (infliximab) or IL-1B are also currently in preclinical trials [29]. By mitigating the pro-inflammatory environment stimulated by the innate immune system, transplanted hair follicles have a chance at survival.

Another avenue to mitigate the rejection of transplanted hairs involves the immune evasion techniques. Investigators are attempting to genetically modify donor follicles to decrease MHC antigen expression to avoid recognition by T cells [30]. Some investigators have suggested encapsulating follicles in immunoprotective biomaterials such as alginate hydrogels [31,32]. This barrier is a physical separation between the follicle and immune cell while allowing for nutrient exchange via blood flow.

Moreover, humanized mouse models have gained traction as the preferred model for preclinical studies. Because they use human immune cells and tissues, they provide a more clinically accurate foundation for studying immune responses to transplanted follicles [33].

Addressing the translational challenges of hair follicle regeneration requires significant advancements in animal studies, in vitro modeling, and immune regulation. By improving our understanding of the cellular mechanisms underlying these obstacles, investigators can design therapies with a higher chance of exhibiting clinical efficacy.

## 3. Wnt/B-Catenin Signaling Pathway

### 3.1. Return in Hair Follicle Neogenesis and Biology

**Figure 1:**
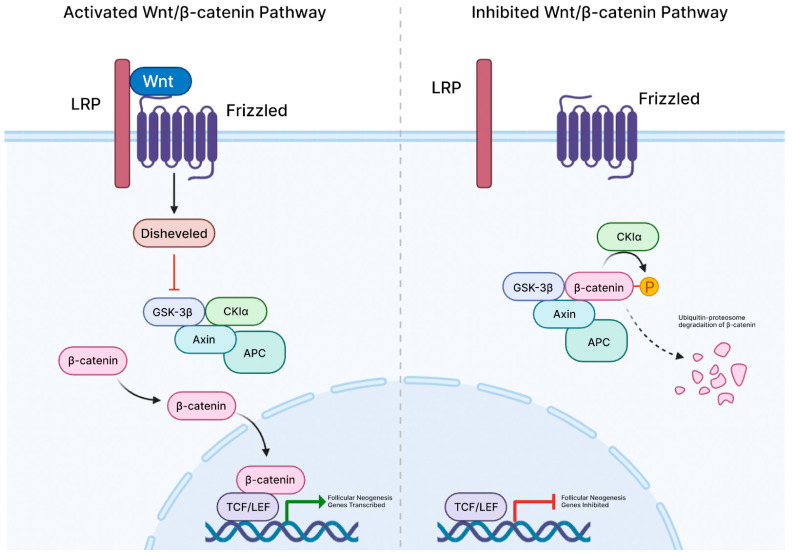
Overview of activated and inhibited Wnt/β-catenin pathway.

Central to hair follicle biology and regulation, the Wnt/ß-Catenin signaling cascade begins with the binding of Wnt to Frizzled receptors and LRP5/6 co-receptors on the cell membrane (Figure 1). This inhibits the ß-catenin destruction complex, which is comprised of APC, Axin, and GSK3ß proteins [34]. This leads to pre-existing ß-catenin stabilizing and translocating to the nucleus, where it binds to the TCF/LEF transcription factors and drives gene transcription that is essential for cellular proliferation, differentiation, and survival. These mechanisms are critical during embryogenesis as well as adult hair follicle regeneration.

During embryogenesis, the Wnt/ß-catenin pathway plays a pivotal role in initiating the formation of a thickened region of the epidermis, which marks the beginning of the hair follicle, known as the hair follicle placode [35]. For example, Huelsken et al. demonstrated that mice deficient in various Wnt signaling components do not complete folliculogenesis, while excessive activation of Wnt/ß-catenin signaling leads to ectopic hair follicle formation [36]. These findings demonstrate the necessity and sufficiency of the Wnt/ß-catenin pathway in driving embryonic folliculogenesis.

The Wnt/ß-catenin pathway similarly plays a key governing role in the adult hair cycle, specifically by regulating the transition from the resting phase (telogen) to the growth phase (anagen). When this pathway is activated, dormant hair follicle stem cells in the bulge region reactivate, leading to increased proliferation and incorporation into the hair matrix [37]. Specialized mesenchymal cells, known as dermal papilla cells, produce many signaling molecules that promote follicular growth and maintenance, and these dermal papilla cells are sustained by Wnt signaling. Ito et al. demonstrated that loss of Wnt signaling resulted in increased telogen length and disrupted anagen initiation in adult mice [38]. Research also suggests that Wnt signaling may be involved with extracellular matrix remodeling by binding to various enzymes, identifying a role for the Wnt/ß-catenin pathway in developing and maintaining an environment conducive to follicular growth [39].

### 3.2. Pathway Regulation

There are many negative feedback pathways through which the proper Wnt/ß-catenin signaling intensity and duration are controlled, among the most important of which include the function of Dickkopf-1 (DKK1) and Secreted Frizzled-Related Proteins (SFRPs) [40]. By competitively binding to the LRP5/6 co-receptors, DKK1 prevents the association of Wnt with these factors, leading to disinhibition of the ß-catenin destruction complex, degrading ß-catenin. Without ß-catenin, gene transcription is not activated for cellular proliferation and survival, leading to decreased growth or even alopecia. In fact, Kwack et al. showed that elevated levels of DKK1 have been correlated with hair follicle miniaturization in androgenetic alopecia [40]. Specifically, it has been shown that DKK1 expression is upregulated in response to DHT, a known correlate of androgenetic alopecia. DKK1 has also been implicated in sebaceous gland function and epidermal homeostasis, both of which are closely tied to hair follicle health.

Another critical negative regulator of the Wnt/ß-catenin pathway is CXXC-type finger protein 5 (CXXC5), which interacts with a cytoplasmic component of the Wnt pathway known as Dishevelled (Dvl) [41]. By binding to Dvl, CXXC5 prevents downstream signaling of the pathway, and elevated levels of CXXC5 are associated with miniaturized hair follicles, thus suggesting that this protein has a role in follicular regression [41]. Lee et al. have shown that targeting the CXXC5-Dvl interaction using small molecule inhibitors can be used to promote hair regrowth by enhancing the activation and activity of the Wnt/ß-catenin signaling pathway, and the interactions between CXXC5 and oxidative stress pathways can also be a potential factor that influences the role of this protein in hair biology [21].

Other signaling pathways impact the role of Wnt/ß-catenin hair growth, the most classical being Bone Morphogenic Protein (BMP) signaling. For example, in separate studies, Kobielack et al. and Choi et al. have shown that BMP signaling ensures that Wnt signaling activation only occurs during the appropriate hair cycle phases (notably anagen) by inhibiting Wnt signaling hair follicle stem cell quiescence [42,43]. Another notable signaling pathway that significantly impacts Wnt/ß-catenin is Sonic Hedgehog (Shh), which supports dermal papilla and enhances hair matrix proliferation by functioning downstream of Wnt/ß-catenin. Lin et al. showed that in adult mice, Shh is upregulated in early anagen, and exogenous application of Shh can prematurely induce anagen in telogenic follicles [44]. They also showed that antibodies blocking Shh’s activity prevented hair growth in adult mice. Notch signaling has also been implicated in intersecting with Wnt/ß-catenin by modulating the differentiation of follicular keratinocytes [45]. Finally, recent studies also underscore the importance of metabolic pathways, such as those that involve AMP-Kinase, linking cellular energy states and hair cycle regulation [46].

### 3.3. Therapeutic Targeting of Wnt/ß-Catenin Signaling

Due to its significant impact on various key aspects of hair growth, Wnt/ß-catenin is viewed as a potential target for therapeutic targets. Based on the desired outcome, this pathway can be enhanced or inhibited. Most focus is placed on the development of therapeutics to activate this pathway to prolong anagen and potentially stimulate hair follicle regeneration.

One promising therapy is peptide-based therapy. For example, PTD-DBM is a peptide therapy that competes with CXXC5 for binding with Dvl [21]. By disrupting this binding, the CXXC5-Dvl complex no longer forms and can, therefore, not disrupt the downstream signaling of Wnt/ß-catenin, ultimately enhancing the effects of this signaling pathway. A study by Lee et al. has shown that applying PTD-DBM topically enhances hair regrowth in mouse models of androgenetic alopecia [21]. This study, among others, highlights the impact of targeting and disrupting the negative regulators of the Wnt/ß-catenin pathway in order to counteract the miniaturization of hair follicles and stimulate regrowth. However, the stability and delivery of these treatments in clinical practice remain challenging.

The converse of inhibiting negative regulators are small molecules that can activate Wnt/ß-catenin signaling by either mimicking Wnt or exogenously stabilizing ß-catenin. For example, by inhibiting GSK3ß, which is part of the ß-catenin destruction complex, the stability of ß-catenin can be drastically increased, which enhances translocation to the nucleus and transcription of relevant genes [47]. While there remain concerns regarding the long-term safety of these treatments, recent clinical trials have reported increased hair density and thickness, although these results are contested [47]. Enhancing the specificity and targeted nature of these therapies will be of utmost importance in future investigations, with novel nanoparticle approaches paving the way.

Finally, supplemental therapies such as low-level laser therapy (LLLT) increase mitochondrial activity and, by proxy, ATP production and ROS signaling [48]. These effects activate the Wnt ß-catenin pathway indirectly and promote hair follicle regrowth. Similarly, physical stimulation methods such as microneedling have recently become popularized as additional approaches to activate the Wnt/ß-catenin pathway [49]. Overall, combining these supplemental therapies with pharmacologic approaches may offer synergistic benefits for hair regrowth [48].

### 3.4. Translational Challenges

While there is significant potential in modulating Wnt/ß-catenin for hair follicle regrowth, several significant challenges remain. Due to the nature of this pathway to promote growth, one of the most prominent and noticeable concerns of this approach is that of off-target effects, as atypical and unnecessary activation of this pathway has been correlated with various cancers such as colorectal and hepatocellular carcinomas [50]. Thus, precise, controlled, and targeted activation of this pathway will be of utmost importance to achieve efficacy while maintaining safety. Current advances in gene-editing technologies such as CRISPR-Cas9 could be achieved in the near future.

Another significant roadblock in utilizing this pathway involves dosage optimization and delivery, as the Wnt/ß-catenin activators only have a narrow therapeutic window. Without enough of these activators, hair follicle regrowth will not be initiated, while an excessive dosage could lead to cancers. Localized delivery may be possible with topical formulations, microneedle patches, or nanoparticle-based delivery systems. Other drug delivery challenges include environmental factors such as pH or temperature [50].

Finally, many of these preclinical studies that demonstrate the efficacy of modulating the Wnt/ß-catenin pathway have been conducted in rodent models. Although a commonly used model system, rodents have noticeably different follicle cycling patterns and exhibit distinct dermal papilla cell behavior compared to humans. These differences can lead to inconsistencies between laboratory findings and clinical outcomes [51]. Because of this discrepancy, it is vital to develop humanized models of hair follicle biology, which can be conducted by using emerging 3D culture systems or organ-on-a-chip technologies to increase translational fidelity.

### 3.5. Emerging Solutions

Significant research is being conducted to improve the safety and therapeutic efficacy of therapies that exploit the Wnt/ß-catenin pathway. Nanoparticle delivery is of particular interest, as nanoparticles can encase the Wnt activators (or the inhibitor deactivators) to protect them from degradation and achieve a more controlled release at the desired site [52].

By optimizing organoid cultures and bioengineered skin solutions, researchers can better test the efficacy of Wnt/ß-catenin-targeted therapies in human models [53]. These models would serve as a stepping stone between preclinical rodent studies and clinical trials for these treatments in humans. Vascular and neural integration of these bioengineered hair follicles is also currently in development, offering a more comprehensive approach to studying this domain.

## 4. JAK-STAT Pathway Inhibitors

### 4.1. Hair Follicle Neogenesis

**Figure 2:**
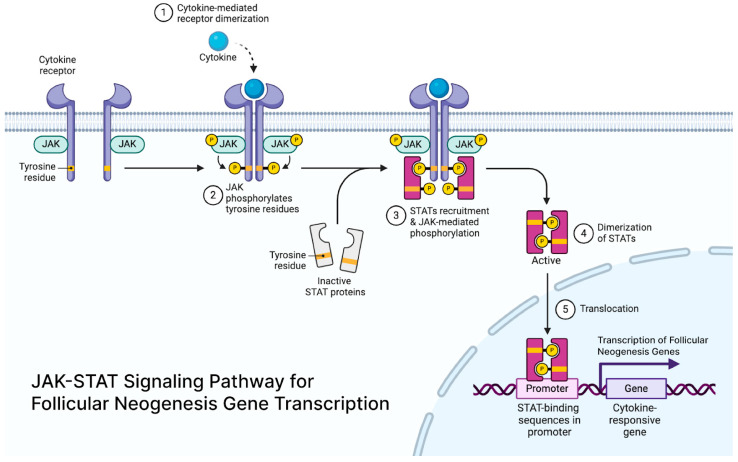
JAK-STAT signaling pathway for follicular neogenesis gene transcription.

JAK-STAT signaling is crucial for maintaining hair follicle stem cells (HFSCs) in a quiescent state, and its inhibition can cause stem cells to enter the growth phase (anagen) of the hair cycle [54] (Figure 2). Extracellular signals like cytokines and growth factors bind to receptors on a family of tyrosine kinases called Janus Kinases (JAKs), causing them to dimerize, autophosphorylate, and then activate. The activated JAK tyrosine residues create docking sites for signaling molecules like STAT proteins, which activate transcription and mediate immune responses [55].

Anagen hair follicles uphold an immune privilege (IP) where they avoid immune-mediated attacks, allowing for continued hair growth [56]. This IP is characterized by decreased stress ligands, decreased major histocompatibility complex (MHC) protein expression, and increased anti-inflammatory cytokines [56]. In a diseased state, hair follicles from alopecia areata (AA) lesions display increased danger ligands; MHC Class I and Class II proteins with self-antigen presentation and pro-inflammatory cytokines like IFN-y and IL-15.56 CD8+ and CD4+ T cells also infiltrate the hair follicle, establishing the characteristic “swarm of bees” appearance in alopecia areata, keeping the HFSC in a state of quiescence, and effectively preventing the anagen stage [57].

The detection of phosphorylated STAT proteins within human and mouse alopecia hair follicles and not in normal follicles indicates that JAK activation is involved in the immune dysregulation in alopecia [58]. Additionally, in scalp and serum biopsies from patients with frontal fibrosing alopecia, a type of scarring alopecia, differentially expressed genes related to CD4+ T helper type 1 (Th1) cells, fibrosis, T-regulatory cells, and JAK were discovered [59]. For this reason, JAK inhibitors are hypothesized to block the activation of this immune response. Mouse studies support this hypothesis by an observed inhibition in the immune response when using the JAK1/2 inhibitor, ruxolitinib, or JAK3 inhibitor, tofacitinib, in vitro within dermal sheath cells from C3H/HeJ mice who develop spontaneous AA similar to human AA [60]. This inhibition of AA development is also seen in vivo within C3H/Hej mice using either JAK inhibitor.

Though scarring alopecia results in a loss of the HFSC niche, resulting in permanent hair loss, JAK-STAT1 inhibitors show promising improvements after mechanistically demonstrating their use by preserving the HFSC niche. In mouse models with a hair follicle-specific epidermal growth factor receptor deletion, a possible mechanism of scarring alopecia, inhibition of JAK1/2 reduced inflammation and restored skin barrier function [61]. This inflammation reduction allows residual stem cells to bolster hair follicle regrowth [61].

### 4.2. Pathway Regulation

Pro-inflammatory signals like IFN-γ and γ-chain cytokines promote quiescence of HSFCs by fueling this JAK/STAT pathway. IFN-γ is activated by immune cells, including but not limited to Th1 and CD8+ T cells. IFN-γ signals through a tetrameric receptor of two IFNGR1 and two IFNGR2 chains, and during immune cell activation, upregulation of IFNGR2 increases sensitivity for IFN-γ and promotes the autophosphorylation of JAK1 and JAK2.56. Studies support the involvement of IFN-γ in AA hair loss by showing a statistically significant increase in serum IFN-γ levels in patients with AA compared to controls [62]. This finding supports the theory that increased IFN-γ levels and other pro-inflammatory signals regulate the JAK-STAT pathway within and around hair follicles to prevent hair growth.

Another pro-inflammatory signal, IL-15, signals through a heterotrimeric receptor with an IL15Rα subunit, a shared IL-2/15Rβ subject, and a γ-chain (γc) subunit [56]. The latter two subunits utilize the JAK-STAT pathway to drive the expansion and maintenance of T cells and natural killer (NK) cells; the shared IL-2/15Rβ subunit binds JAK1, while the γc subunit binds JAK3.56. AA patients also had increased serum levels of IL-15 compared to controls, and even more in patients with alopecia totalis, a severe form of alopecia areata [63].

### 4.3. Therapeutic Inhibition of JAK-STAT

First-generation JAK inhibitors include baricitinib, ruxolitinib, and tofacitinib, all of which target JAKs or cytokines involved in the JAK-STAT pathway. A meta-analysis of two retrospective and two prospective studies on oral ruxolitinib and baricitinib displayed that both JAK inhibitors significantly increased the frequency of good responses (defined as a >50% decrease in Severity of Alopecia Tool (SALT) score) compared to the placebo group [64]. In June 2022, the Food and Drug Administration (FDA) approved a wide-scale JAK inhibitor clinical trial of baricitinib. A retrospective study of the JAK 1 and 2 inhibitor, baricitinib, demonstrated that when taken at 4 mg orally once daily, 59 out of 96 patients achieved a SALT score of ≤20 in 52 weeks when, at baseline, the mean SALT score was 84.5 (SD 18.6) [65]. Separately, in two phase three trials of baricitinib, the percentage of patients who achieved a SALT score of ≤20 in 36 weeks was 38.8% with 4 mg baricitinib, 22.8% with 2 mg baricitinib, and 6.2% with a placebo in the first trial, and 35.9%, 19.4%, and 3.3%, respectively, in the second trial [66].

A retrospective study of patients with moderate-to-severe AA concluded that combining tofacitinib and systemic corticosteroids (SCs) treated hair loss, with 78.3% of patients achieving a SALT score of 50. In total, 60.0% of patients achieved a SALT score of 50 in the tofacitinib-only group, and 66.7% of patients achieved SALT_50_ in the SC-only group [67]. This study showed that supplementing SC with tofacitinib or use of tofacitinib alone could be an effective treatment for AA while reducing the number of adverse events that accompany long-term steroid use [67]. Within a systematic review of nine studies evaluating JAK inhibitors for the treatment of cicatricial alopecia (CA), a type of scarring alopecia, tofacitinib was a safe and effective treatment for CA [68]. Minimal adverse events occurred; however, recurrence of hair loss after cessation of JAK inhibitor treatment was observed in four of the nine studies [68].

### 4.4. Translational Challenges

The use of JAK inhibitors for inflammatory alopecias can lead to systemic immunosuppression and lab abnormalities. Although well tolerated, a systematic review of adverse events (AEs) in studies involving JAK inhibitors for AA included abnormal lab results (40.1% of the total number of AEs) involving hepatic and metabolic panels, especially elevated cholesterol. The remaining AEs involved upper respiratory tract infections, acne, urinary tract infections, and diarrhea [69]. Despite the evidence of mild AEs with the use of JAK inhibitors, a meta-analysis of the safety and efficacy of JAK inhibitors showed that the incidence of AEs was significantly lower with oral JAK inhibitors than with SCs [64]. This evidence suggests that JAK inhibitors can be a safer option for the treatment of inflammatory alopecias.

Since scarring alopecia is seen as a permanent hair loss issue, there are challenges in assessing the use of JAK inhibitors for subtypes of alopecia outside of AA. There is typically little to no hair regrowth in these scarring alopecia patients, whereas the success of JAK inhibitors is visible in alopecia areata patients who experience hair regrowth. Significant challenges limiting hair follicle neogenesis in scarring alopecia are fibrosis and destruction of the stem cell niche. During the inflammatory process of scarring alopecia, the normal hair follicle is replaced with scar tissue, and this new environment is not conducive to neogenesis [70]. Even if JAK inhibitors reduce or prevent the inflammatory process and preserve the IP of HFSCs, ones already replaced with fibrous tissue may not be able to regenerate with JAK inhibitor use.

## 5. Emerging Therapies Beyond Wnt Signaling and JAK-STAT Modulation

In recent years, there has been significant progress in understanding the molecular mechanisms that underlie hair follicle regeneration and alopecia. While JAK-STAT pathways and Wnt signaling are key areas of study, new emerging therapies now explore alternative options and novel targets, overcoming the limitations of the existing treatments. Advancements in stem cell-based approaches, growth factor delivery, gene editing, and prostaglandin analogues are the new frontier of hair restoration therapies.

### 5.1. Stem Cell Approaches

Stem cell therapy and hair tissue engineering are novel approaches to treating hair loss. These strategies create hair using a tissue-engineering approach, reverse the pathological mechanisms leading to hair loss, and regenerate hair follicles [71].

#### 5.1.1. iPSCs for Follicular Regenerations

Pluripotent stem cells (iPSCs) reprogrammed from patients’ cells have potential for patient-specific therapy. iPSCs can differentiate into multiple cell types, making their utilization optimal for drug development, disease research, and regenerative medicine [72,73]. Their pluripotent potential make them ideal for hair follicle regeneration. Use of native cells would also reduce the risk of immune rejection and other complications often linked to traditional treatments [72]. The potential of iPSCs has been highlighted as a limitless source of hair follicles for transplantation to treat hair loss since they can be used in human hair follicle regeneration [74].

#### 5.1.2. Autologous Cell Grafts

It is also important to briefly mention autologous cell micrografts (ACMs), which have become a conventional treatment technique for androgenetic alopecia (AGA). Although progenitor cells are damaged in AGA, human hair follicle stem cells (HFSCs) are preserved allowing for the ACM method to capitalize on this multipotency. Gentile et al. demonstrated ACM using Rigeneracons (CE certified class I) to disaggregate and centrifuge punch biopsies of patients’ scalps, effectively isolating mesenchymal stem cells CD44+ and CD200+ within a cell suspension [75]. This method allowed for the patient’s own HFSCs to be immediately injected back into their scalp with the use of a culture medium. This treatment led to a 29% ± 5% increase in hair density for 11 patients (38 to 61 years old) affected by AGA [75]. Additionally, a retrospective study evaluated 140 adults with AGA and found an increase in hair density, average hair thickness, percentage of thick hair, number of follicular units, and cumulative hair thickness in 66.4% of participants who underwent ACM, especially within the frontal scalp regions [76].

#### 5.1.3. Dermal Papilla-like Stem Cells

Dermal papilla cells (DPCs) are a physical and chemical reservoir for epithelial progenitor cells that aid the regeneration of the cycling portion of the hair follicle and generate the hair shaft [75]. DPCs are located at the base of hair follicles, where they control the cycling of the hair follicle and are essential contributors to hair follicle homeostasis [76]. Therefore, there has been a push to reprogram stem cells to mimic the dermal papilla stem cells. Investigators have succeeded, primarily in mice models, in differentiating pluripotent cells into dermal papilla-like cells that mimic their characteristics [76]. For instance, Riabinin et al. differentiated human pluripotent stem cells into neural progenitor cells and subsequently into dermal-papilla-like cells in mice. These newly differentiated cells expressed dermal papilla cell markers and induced early stages of folliculogenesis in vitro [76]. These could be useful tools for hair follicle bioengineering and lead to novel therapies for scalp and hair diseases.

Recent studies have shown that extracellular vesicles (EVs), cell-derived nanoparticles (CDVs), and engineered nanovesicles (eNVs) can be used as effective therapeutic arms in hair regeneration as drug delivery vehicles. Improvements in EV technology have allowed researchers to engineer EV mimetics as an alternative biomaterial in translational medicine [77]. EVs can be naturally released from cells into their extracellular environment, where they can communicate with the surrounding cells to alter their biology. It has been seen that released EVs from cells in cell culture can be isolated and used as effective therapeutic agents [78,79]. Dermal papilla cell-derived EVs, stem cell-derived EVs, and neural progenitor CDVs studies are emerging as key therapeutic arms in hair regeneration [80,81,82]. EVs and eNVs exhibit their therapeutic effects by interacting with surrounding cells or releasing their intravesicular content after they are internalized. It has been shown that various intravesicular cargoes can activate signaling pathways to induce hair growth in vitro, ex vivo, and in vivo [76].

Stem cell therapy use in regenerative medicine, such as for hair loss therapy, encounters practical issues that limit their efficacy as effective therapeutics, including immunogenicity, tumorigenicity, and heterogeneity [53]. Immune rejection has always been a critical issue in cell therapy as the immune system recognizes transplanted stem cells as foreign attacks, rejecting the grafted cells [83]. There has been an effort to overcome these challenges through HLA matching, yet it continues to limit the use of stem cells as a regenerative therapy for hair loss. Tumorigenicity caused by reprogramming factors represents a significant concern. The four reprogramming factors have all been related to tumorigenicity, such as c-Myc, which functions as the driver mutation for many of these iPSCs and is one of the most frequently mutated genes in human cancers [84]. Additionally, teratoma formation is another significant concern for hiPSCs and hESC cell transplantation. If lineage-specific stem cells exist within the transplant, then tumors can form because of incorrect or incomplete patterning [85].

### 5.2. Growth Factor Delivery (VEGF, IGF-1, FGF-7)

Growth factor-based therapeutics are an emerging promising approach to hair restoration. Growth factors such as vascular endothelial growth factor (VEGF), insulin-like growth factor-1 (IGF-1), and fibroblast growth factor-7 (FGF-7) play crucial roles in angiogenesis, follicular proliferation, and the hair growth cycle. Developing innovative delivery systems such as microneedles, hydrogels, and nanocarriers has helped enhance the bioavailability, stability, and targeted release of these factors to address challenges such as limited follicular penetration and rapid degradation.

#### 5.2.1. Mechanism

These growth factors act on several aspects of the hair growth cycle to improve hair regeneration in novel therapies. Platelet-derived growth factor (PDGF) helps enhance hair growth and stimulates vascularization and angiogenesis to boost hair growth. PDGF can also help the chemotaxis and proliferation of fibroblasts and the mitogenesis of mesenchymal stem cells and endothelial cells [71]. Dermal papilla cells express VEGF during the anagen phase of hair growth, and VEGF plays a critical role in controlling perifollicular angiogenesis [71]. In addition, it can enhance the size of perifollicular vessels, facilitating the uninterrupted continuation of the anagen growth phase. In addition, FGF-7 functions by acting on hair growth through cell multiplication to prolong the anagen stage. Epidermal growth factor (EGF) stimulates the growth and division of both mesenchymal and epithelial cells and mediates hair cell proliferation and regeneration [86]. Moreover, it acts with other factors to promote angiogenesis, fostering the development of new blood vessels. IGF-1 controls the growth of hair follicles by working as a stimulator for angiogenesis and increasing overall hair growth [87]. All these growth factors function by acting on the hair follicle stem cells, ultimately promoting neovascularization and new follicle growth. Overall, IGF-1 stimulates follicular growth, EGF stimulates the proliferation of the hair, FGF-7 prolongs the anagen stage, PDGF constructs the hair canal, and VEGF stimulates angiogenesis of the hair shaft.

#### 5.2.2. Evidence from Trials

Platelet-rich plasma (PRP), a treatment that uses a patient’s platelets to promote healing, has become one of the budding techniques for tackling hair loss. PRP is rich in the aforementioned growth factors, making it a suitable treatment to administer. Its ability to act on hair follicles makes PRP an increasingly attractive therapy in regenerative medicine, and different clinical investigations have shown promising outcomes concerning hair regeneration and growth. A 2019 study showed that hair density and count in patients with scalp alopecia significantly improved the PRP-treated scalp group compared with the placebo-treated scalp group [88]. In addition, PRP was even found to be well tolerated and effective against hair loss [88]. In another study looking at PRP’s impact on individuals with alopecia areata, PRP reduced the number of dystrophic and vellus hairs with complete remission in 60% of patients and induced hair growth [89]. Finally, PRP has been recommended in one review to be used synergistically with other conventional therapies such as finasteride, spironolactone, and minoxidil to improve outcomes [90].

#### 5.2.3. Challenges

Because of the novelty of using growth factors to treat hair loss, some challenges are associated with their implementation. Extraction of these growth factors is mainly limited to extracting the patient’s plasma. The research on these growth factor treatments for hair loss is limited, and more robust clinical studies are needed to obtain true efficacy [86]. There is individual variability as the response to the growth factor dramatically varies between individuals. Finally, multiple treatment sessions are required to increase effectiveness, which is costly and time-consuming [86].

### 5.3. Gene Editing and CRISPR

Emerging research highlights CRISPR/Cas9’s role in editing key genes for hair growth. These findings promise advancement in understanding the genetic causes of hair loss and the potential to develop personalized therapies.

#### 5.3.1. Mechanism

CRISPR/Cas9 is a highly sophisticated gene-editing technology that precisely modifies DNA by utilizing a naturally occurring bacterial defense mechanism. The system consists of the Cas9 enzyme, which acts as molecular scissors, and a guide RNA (gRNA) that directs Cas9 to a specific DNA sequence. When the gRNA binds to its complementary target sequence within the genome, Cas9 will induce a double-strand break at that site [91]. The cell then attempts to repair the double-strand break, which allows for precise gene modifications by incorporating a provided DNA template. This particular and programmable system is now being used to edit genes related to hair growth and hair cycle regulation in multiple recent studies exploring the significance of modulating different molecular pathways [92].

Studies on the CRISPR/Cas9 modulation of different genes can be categorized into studies with cashmere goats, rabbits, and C57BL/6 and BALB/c mice.

CRISPR/Cas9 has been used to edit three genes associated with hair growth in cashmere goats: *FGF5*, *Tβ4*, and *VDR*. FGF5 functions by stimulating hair cycle activation, which has been shown to increase fiber length and the amount of hair follicles [93]. Tβ4 acts via increasing angiogenesis, vascular permeability surrounding the area of the secondary hair follicles, and vasoconstriction. These combined effects have been shown to increase hair length and the amount of hair follicles [94]. Finally, editing the *VDR* gene suppressed the expression of Noggin, LEF1, β-catenin, and VGF and the BMP4 and WNT pathways. Researchers subsequently saw that this significantly decreased growth impacted hair formation and halted the growth cycle [95]. Finally, *FGF3* was targeted by CRISPR/Cas9 in rabbits, which led to a suppression of the VERSICAN (an extracellular matrix proteoglycan that modulates key signaling pathways like Wnt and BMP to regulate hair follicle development) signaling pathway and BMP2/4 pathway. This resulted in an increased fiber diameter, an increased number of follicles per area, and the prolongation of the anagen phase of the hair cycle and the shortening of the other hair cycle phases [96].

*SRD5A2*, *FGF5*, *PLCD1*, and *CCHCR1* are edited genes linked with hair regeneration in C57BL/6 mice. When edited, *SRD5A2* increased VEGF expression but suppressed apoptosis, leading to an increase in the amount of hair follicles and hair follicle thickness [97]. Like in cashmere goats, the *FGF5* gene in C57BL/6 mice was edited, which was shown to induce the activation of the WNT/β-catenin signaling pathway. The activation led to the increased thickness of hair follicles, amount of follicles, and hair length [98]. PLCD1 impacted the FOXN1 signaling pathway and affected the genes involved with differentiation of the hair follicle, which created baldness on the mouse’s abdomen, decreased the number of hair follicles, changed epidermal differentiation, and caused histological abnormalities [99]. Finally, CCHCR1 was shown to be involved with hair development and the keratinization signaling pathway, which was linked to morphological abnormalities and hair loss [100]. In BALB/c mice, FGF21 is a studied target that caused suppression of the MAPK/ERK and PI3K/AKT signaling pathways. When suppressed, these pathways decrease the amount of hair per area and the rate of hair regeneration [101]. Finally, in both C57BL/6 and BALB/c mice, LAMA3 was targeted, which led to the suppression of the PI3K/AKT signaling pathway that had downstream effects of increasing the size of sebaceous glands, a lack of the typical hair follicle structure, and hair loss 80 days after birth [102].

#### 5.3.2. Challenges

There are some barriers surrounding CRISPR/Cas9’s clinical implementation for safe and effective use for hair loss. Despite the precision of CRISPR/Cas9, there is a need for precise genetic editing as off-target mutations pose a significant concern for many researchers. Therefore, further research needs to be performed on the long-term effects of CRISPR/Cas9 before clinical trials can be safely applied to humans [92]. In addition, researchers need to factor in accessibility, public perception, and ethical and regulatory concerns. Finally, there needs to be a decrease in the adverse immune responses induced by this technique, and the development of effective delivery systems needs to be improved [92].

### 5.4. Prostaglandin Analogues

Prostaglandins are bioactive lipids crucial in regulating hair follicle cycling and growth. Prostaglandin D2 (PGD2) and prostaglandin E2 (PGE2) are two prostaglandins that have garnered significant attention for their opposing effects on hair follicles. PGD2 acts as a hair growth inhibitor, while PGE2 promotes follicular proliferation and regeneration. Prostaglandin-based therapies, including latanoprost and bimatoprost, have shown promise in stimulating hair growth, particularly in conditions like androgenetic alopecia and hypotrichosis. However, despite prostaglandin analogues’ potential, their limited efficacy in alopecia areata and other hair loss disorders, paired with challenges in drug delivery and long-term response, serves as a significant hurdle to its translation to the bedside.

#### 5.4.1. Overview of Prostaglandins

PGD2 and PGE2 have been seen to have opposite effects on hair growth as PGD2 acts as a hair growth inhibitor, and PGE2 has been seen to enhance hair growth. PGD2 has been shown to be elevated in individuals with androgenetic alopecia. Research indicates that PGD2 inhibits hair growth by binding to the G protein-coupled receptor 44 (GPR44), also known as the DP2 receptor. Activating this receptor leads to the suppression of hair follicle elongation and promotes the transition from the anagen phase to the catagen phase of the hair cycle [41]. Conversely, PGE2 promotes hair growth by stimulating hair follicle proliferation and prevents hair loss from different factors. One of these factors includes protection from radiation-induced hair loss in mice, which suggests that it has a role in maintaining hair follicle health and promoting regeneration [103].

#### 5.4.2. Latanoprost and Prostaglandins

Johnstone proved latanoprost to be an effective hair growth stimulant. This study showed that with latanoprost use, an increased amount of hair, length, thicker follicles, hair curvature, and pigmentation were observed in the latanoprost-treated eye [104]. Additionally, a placebo-controlled trial in men with mild androgenetic alopecia (AGA) revealed that the application of latanoprost significantly augmented pigmentation and hair density [105]. Men with AGA were treated with latanoprost and a placebo on two zones of the same scalp. This study illuminated that the latanoprost-treated sites showed increased hair density compared with the placebo [105].

Additionally, bimatoprost is a Food and Drug Administration (FDA)-approved PGF2α analogue for eyelash hypotrichosis and can be used for other hair disorders. These other hair disorders include eyebrow hypotrichosis, chemotherapy-induced hypotrichosis, and eyebrow thinning. One study highlighted that bimatoprost is as effective as minoxidil with fewer side effects in terms of enhancing eyebrow thickness. A separate double-blind, parallel-group, randomized, and multicenter study showed that treatment of a bimatoprost ophthalmic solution daily for a year was well tolerated in patients with chemotherapy-induced and idiopathic eye hypotrichosis [106].

#### 5.4.3. Translational Challenges

Translational challenges have required these treatments for hair loss to be studied more extensively to understand their mechanisms better. A one-year retrospective study shows that bimatoprost has had little to no efficacy when treating AA [107]. Another randomized, controlled, investigator-masked study showed that eyelash AA patients treated with topical latanoprost and bimatoprost ophthalmic solutions exhibited no significant changes [108]. Latanoprost and bimatoprost use have been associated with various local and systemic side effects, such as eye irritation, dry eye symptoms, conjunctival hyperemia, and eye pruritus [109,110,111]. Despite multiple studies and clinical reports of prostaglandin-induced hair growth, their mechanisms of action are still unknown.

## 6. Conclusions

The profound psychosocial impact of alopecia emphasizes the necessity of developing practical, targeted therapies to treat the disease. Though traditional treatments such as minoxidil and finasteride have shown to be beneficial for some patients, their application across various types of alopecia presents significant limitations, accompanied by significant side effects. Strides made in molecular biology and our understanding of signaling pathway modulation have presented us with an opportunity to advance hair restoration therapies, such as those emphasized in this review: hair follicle neogenesis, Wnt/β-catenin modulation, JAK-STAT inhibitors, stem cell-based approaches, gene editing, and more.

Among these, Wnt/β-catenin pathway modulation has remained in focus as the central pathway to stimulate hair follicle regeneration, with therapies such as PTD-DBM and GSK3β inhibitors presenting the most tangible outcomes in preclinical models. Additionally, JAK inhibitors such as ruxolitinib and tofacitinib have revolutionized alopecia areata treatment through immune system modulation and restoration of the hair follicles’ immune privilege. Stem cell-based therapies and bioengineered hair follicles are also being explored, though they face translational obstacles such as immune rejection and accurate in vitro replication in human-made microenvironments.

Despite significant progress in hair restoration therapies, existing challenges prevent adoption in clinical settings. Challenges that must be addressed for this widespread adoption to occur range from limitations in preclinical models, safety and tumorigenicity, limitations in precise delivery, and hair follicle biological complexity. Future studies should optimize targeted drug delivery in preclinical models and present a path forward for translating scientific findings into clinical care.

Ultimately, researchers and clinicians have realized that treating alopecia requires a multipronged approach integrating molecular biology, regenerative medicine, and targeted immunotherapy. Rigorous investigation will be necessary to refine emerging therapies, ensuring they are clinically effective and carry a safe risk profile. As we learn more about hair follicle microbiology and treatments, the next phase of hair restoration will depend on transferring our findings from preclinical models to clinical settings. With advancements made thus far, alopecia reversal and treatment in a safe, personalized, and effective manner is becoming increasingly within reach.

## Figures and Tables

**Table 1:** Comparison of Traditional and Emerging Therapies.

Treatments	Indications	Strengths	Limitations
Traditional Therapies: Finasteride (oral) and Minoxidil (topical/oral)	- Finasteride: FDA-approved for androgenetic alopecia (AGA) in men - Minoxidil: FDA-approved for AGA in men and women	- Demonstrated efficacy - Finasteride: once daily oral dosing - Minoxidil: easily accessible topical and oral formulation; oral serves as step-up therapy	- Continuous use required - Oral use may lead to unwanted facial or body hair growth - Clinical efficacy and risk profile are not consistent across patients - Finasteride: potential adverse sexual side effects including erectile dysfunction and decreased libido - Limited in efficacy beyond AGA
Wnt/β-Catenin Modulation: PTD-DBM, GS3KB, and Low-Level Laser Therapy	- No official approval - Seen in mouse models to enhance hair growth in AGA through topical application	- Would allow for direct modulation of an established central hair follicle neogenesis pathway	- Expected off-target effects -> atypical activation of the pathway associated with colorectal and hepatocellular carcinomas - Narrow therapeutic window in Wnt/β-Catenin activation - Majority of work in rodent models
JAK-STAT Inhibitors: Baricitinib, Ruxolitinib, Tofacitinib, Ritlecitinib	- FDA-approval of baricitinib, ritlecitinib, deuruxolitinib for alopecia areata (AA)	- Strong responses in alopecia areata trials - Synergistic response with tofacitinib + corticosteroids - Meta-analysis showed better adverse effect profile than corticosteroids	- Immunosuppression and adverse effects: elevated hepatic/metabolic panels (elevated cholesterol), URI, acne, UTI, diarrhea - Limitations in scarring alopecias and other subtypes of alopecia
Stem Cell Approaches: iPSCs, DPCs, Autologous Grafts	- No official approval - Experimental treatments	- Pluripotency gives potential for patient-specific-therapy - iPSC to DPC differentiation leading to early folliculogenesis seen in vitro	- Immune rejection as a core issue - Tumorigenicity caused by reprogramming factors such as c-Myc, a common cancer mutation - Teratoma formation risk
Growth Factor Delivery Systems: VEGF, IGF-1, FGF-7	- No official approval - Experimental treatments through platelet-rich plasma (PRP) delivery	- Some results show complete remission and hair growth in alopecia areata - Recommended in a review as synergistic with traditional therapies	- Limited research on the effects of these growth factor treatments - Significant individual variability in response to growth factors - Multiple treatment sessions needed
Gene Editing: CRISPR/Cas9	- No official approval - Experimental treatments	- Modulation of genes in cashmere goats, rabbits, C57BL/6 mice, and BALB/c mice demonstrated ability to increase or reduce hair loss through gene-dependent mechanisms	- High need for precision: off-target mutations pose a significant concern - Limited research on long-term effects of CRISPR/Cas9 - Accessibility, public perception, and ethical/regulatory concerns - Need to decrease adverse immune response
Prostaglandin Analogs: Latanoprost and Bimatoprost	- Bimatoprost FDA-approved for eyelash hypotrichosis - Experimental for AGA and AA	- Bimatoprost demonstrated to be as effective as minoxidil with fewer side effects for eyebrow hypotrichosis - Latanoprost showed promise in mild AGA	- Limited efficacy in alopecia areata and other hair loss disorders - Local and systemic side effects: eye irritation, dry eye, conjunctival hyperemia, and eye pruritis - Unclear mechanisms of action

## Data Availability

Not applicable.
